# Resistance to change: AMR gene dynamics on a commercial pig farm with high antimicrobial usage

**DOI:** 10.1038/s41598-020-58659-3

**Published:** 2020-02-03

**Authors:** Jolinda Pollock, Adrian Muwonge, Michael R. Hutchings, Geoffrey Mainda, Barend M. Bronsvoort, David L. Gally, Alexander Corbishley

**Affiliations:** 1 Animal and Veterinary Sciences, Scotland’s Rural College (SRUC), Edinburgh, United Kingdom; 2 The Roslin Institute and Royal (Dick) School of Veterinary Studies, University of Edinburgh, Edinburgh, United Kingdom

**Keywords:** Antimicrobial resistance, Microbiome

## Abstract

Group antimicrobial administration is used to control disease in livestock, but we have little insight into how this impacts antimicrobial resistance (AMR) gene dynamics. Here, a longitudinal study was carried out during a single production cycle on a commercial pig unit with high historic and current antimicrobial usage. Quantitative PCR, 16S rRNA gene metabarcoding and shotgun metagenomic sequencing were used to track faecal AMR gene abundance and diversity and microbiome alpha diversity. Shotgun metagenomic sequencing identified 144 AMR genes in total, with higher AMR gene diversity present in young pigs compared to dry sows. Irrespective of in-feed antibiotic treatment or changes in microbiome diversity, mean AMR gene copy number was consistently high, with some AMR genes present at copy numbers comparable to the bacterial 16S rRNA gene. In conclusion, AMR gene prevalence and abundance were not influenced by antibiotic use, either during the production cycle or following whole-herd medication. The high levels of certain genes indicate they are widely disseminated throughout the microbial population, potentially aiding stability. Despite the high and relatively stable levels of resistance genes against the main antimicrobials used, these compounds continue to control production limiting diseases on this unit.

## Introduction

Antimicrobial agents are used regularly in many agricultural systems worldwide to improve the health, welfare and productivity of livestock^1,2^. This has led to concerns about the anthropogenic selection of antimicrobial-resistant bacteria in livestock systems^2–4^, particularly due to the potential transfer of antimicrobial resistance (AMR) genes from livestock to humans^5–7^ and into the environment^8,9^. In the United Kingdom, 52% of all antimicrobials sold for livestock were used in the pig and poultry sectors, with tetracyclines being reported as the highest sold antibiotic class^10^. Given the substantial use of antimicrobials in pig production, the association between antimicrobial use and AMR has been an area of intensive study. Specifically, antimicrobial administration has been associated with increased AMR in sentinel bacteria such as *Escherichia coli*^11–13^, *Staphylococcus aureus*^14,15^, and *Campylobacter*^16^, *Salmonella*^17^ and *Enterococcus*^18^ species. Molecular studies have revealed a higher richness and diversity of AMR genes in pigs administered oxytetracycline in-feed^19^, with farm origin being associated with AMR gene abundances in faeces at slaughter using both quantitative PCR^20^ and metagenomic^21^ datasets. However, there are currently few longitudinal studies of AMR in livestock systems, specifically for pig production^22^, with information on antimicrobial use in these systems often derived from national figures, rather than from farm medicines records^12,21^.

Here, we examine microbial population dynamics and both AMR gene abundance and diversity on a single pig farm during a six-month production cycle. Faecal samples were collected weekly from a batch of young pigs that were followed from birth to slaughter. Group treatments during this time included acidified water, dietary zinc supplementation and a prolonged period of in-feed chlortetracycline, followed by tylosin administration. Faecal samples from dry sow accommodation containing unmedicated pigs were taken in parallel. Finally, faecal samples were taken from dry sows during and after a partial depopulation event which involved antibiotic administration to every pig on the farm to assess if this practice impacted on AMR gene abundance. Microbiome alpha diversity, AMR gene abundance and AMR gene diversity were measured by 16S rRNA gene metabarcoding, quantitative PCR (qPCR) and shotgun metagenomic sequencing, respectively.

## Materials and Methods Study Farm

In order to study the impact of antimicrobial usage on AMR gene abundance and diversity, a 600 sow Landrace x Large White commercial farrowing to finishing unit (United Kingdom) with high historic and current antimicrobial usage was recruited. In the three months prior to the study period, a total of 389.1 mg/Population Correction Unit (PCU) of antibiotics were used on this farm. This compares to a UK average in pigs of 183 mg/PCU in 2016 and 131 mg/PCU in 2017, making this a high antibiotic usage farm^10^. Detailed antimicrobial usage records and additional herd information are available in Supplementary Materials 1. This study received ethical approval from the Royal (Dick) School of Veterinary Studies Veterinary Ethics Research Committee. All methods were conducted in accordance with institutional and national guidelines and regulations.

### Sampling

Faecal samples were taken from this unit during two time periods (Phase 1 and Phase 2) as described below. All samples were collected in universal tubes and stored at −20 °C on-site, prior to batch transportation to the laboratory on dry ice and storage at −80 °C until processing.

#### Phase 1: Faecal sampling during a single production cycle

**Figure 1:**
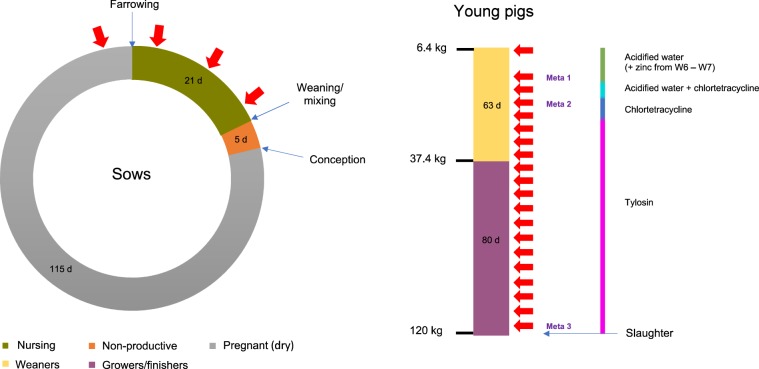
Summary of pig production cycle, antimicrobial administration and sampling points. Faecal sampling (red arrows) was carried out from farrowing pens containing the pregnant sows only (W1), and then post-farrowing from both the sow and the nursing pigs during co-housing (W2-W4). Weaners were mixed and moved into new accommodation prior to sampling on W5 and were moved to larger accommodation for the growing/finishing period prior to sampling on W14. Samples subject to metagenomic sequencing are indicated (Meta 1 to Meta 3). No sampling was carried out on W6 due to access issues.

Pooled faecal samples from the pen floors containing medicated young pigs (referred to as “young pig accommodation”) were collected weekly from just prior to farrowing on 26^th^ October 2016 to 5^th^ April 2017 (W1 to W25), when the studied batch were sent for slaughter. The sampling design and periods of in-feed medication are summarised in Fig. 1. Weekly faecal samples were also taken randomly from the dry sow barn floor (referred to as “dry sow accommodation”) (n = 6) to establish background levels of AMR genes in a group of adult pigs that were not routinely medicated with antimicrobials. Further medication and sampling details are available in Supplementary Materials 1.

#### Phase 2: Faecal sampling during and after a partial depopulation

In an effort to improve health status and reduce antimicrobial use, a partial depopulation was carried out on this farm in May 2017. This involved removing all the young pigs (i.e. nursing piglets, weaners, growers and finishers) from the unit, fumigating the young pig accommodation with formaldehyde and administering in-feed chlortetracycline and tiamulin to all the breeding sows for a period of six weeks. In order to establish the impact of this management practice on AMR gene abundance and diversity, faecal samples were obtained from the dry sow accommodation during the final week of antibiotic administration (W36), two months after treatment (W45) and five months after treatment (W57) (n = 6).

### Sample preparation and analysis

#### DNA extractions and dry matter calculations

DNA extractions were carried out using the DNeasy PowerSoil Kit (Qiagen, UK) following the manufacturer’s instructions. In parallel, approximately 1 g of homogenised faeces were weighed out and placed into a drying oven overnight at 60 °C. After re-weighing, the percentage of dry matter (% DM) was determined.

#### Quantitative PCR

Five AMR genes associated with the main antibiotics used on the unit were targeted for longitudinal analysis based on initial end point screening of 36 AMR genes. These were *tetB* and *tetQ* (tetracycline), *ermA* and *ermB* (tylosin) and *dfrA1* (trimethoprim). Additionally, quantification of the 16S rRNA gene was included as a proxy of overall bacterial load. Plasmids containing gene fragments of *tetB*, *tetQ*, *ermA*, *ermB*, *dfrA1* and the 16S rRNA gene were generated in-house and used as standards for absolute quantification of gene copy number per gram of dry faeces. Further details on methodology and primer/probe sequences are shown in Supplementary Materials 1 and 2.

#### 16S rRNA gene metabarcoding

All DNA extracts were prepared for 16S rRNA gene metabarcoding targeting the V3 hypervariable region, as described in previous work^23^ and in Supplementary Materials 1. The sequence files generated (Edinburgh Genomics, UK) with the primers removed, are publicly available through the NCBI Sequence Read Archive (SRA) under the BioProject accession number PRJNA557844. Using a mock bacterial community (20 Strain Even Mix Genomic Material ATCC®MSA-1002, ATCC, United States), the mean sequencing error rate was calculated as 0.01%.

The generated sequences were processed using cutadapt^24^ and mothur^25^ (URL: https://www.mothur.org/wiki/MiSeq_SOP. Accessed January 2018) as described previously^23^. Here, unique sequences were binned into operational taxonomic units (OTUs) using a database-independent approach. A mean of 125,312 sequences per sample were retained after quality control and were subsampled to 10,000 sequences per sample for analysis. Both the Inverse Simpson index (ISI) and Shannon index (SI) were calculated for each sample to assess alpha diversity.

#### Shotgun metagenomic sequencing

Three time points were selected that corresponded to differing antimicrobial treatment phases in the young pigs (Fig. 1). Triplicate samples from both the dry sow and young pig accommodation (n = 18) were submitted for shotgun metagenomic sequencing (Edinburgh Genomics, UK) with further details on library preparation and analysis^26–34^ available in Supplementary Materials 1. The sequence files are publicly available on the European Nucleotide Archive (ENA) under study accession number PRJEB34736.

#### Statistical analysis of alpha diversity and gene abundance data

Temporal changes in both gene copy number and alpha diversity indices were assessed statistically using repeated measures analysis of variance (Genstat 16, VSN International, UK) with the data obtained from the dry sow and young pig accommodation faecal samples. To assess the impact of the antimicrobial treatments on gene copy number and alpha diversity indices in the young pig accommodation, least significant differences were used for multiple comparisons of means.

For the dry sow analyses, the values calculated from samples collected between W1-W25 were included in the statistical models. For the young pig analyses, the values calculated from samples collected between W5-W25 were included in the statistical models, since W1 samples were obtained from pregnant sows only and samples obtained between W2-W4 were obtained when the piglets were still grouped by litter and were not yet assigned to their rearing pens. For the young pig analyses, the pen was included as a factor to assess any differences between the triplicate pen samples.

## Results

### AMR gene abundances during a full production cycle

**Figure 2:**
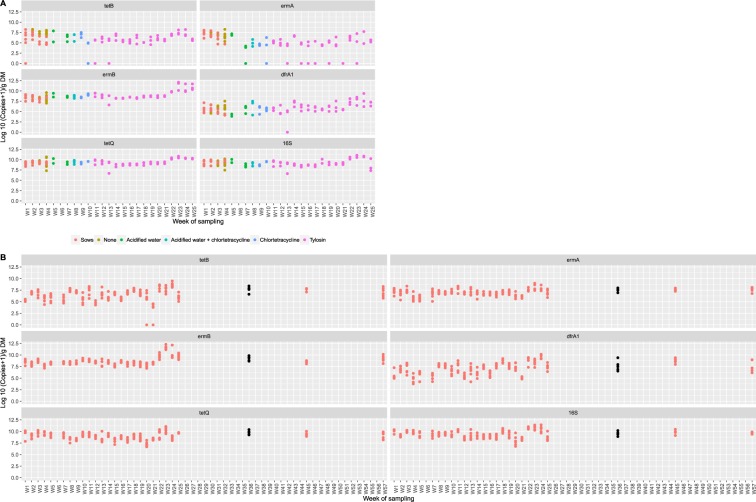
Quantification of AMR genes. AMR gene copy numbers in faecal samples obtained from the (**A**) young pig and (**B**) dry sow accommodation including the partial depopulation. During the study period, the following routine group medication regimens were used in the young pigs: toltrazuril (30 mg/head oral) at 4 days old, zinc (2500 ppm in feed) between 4 and 6 weeks old, acidified water (Baynes Evacide S 0.2%) between 3 and 7 weeks old, chlortetracycline (300 ppm in feed) from 6 to 8 1/2 weeks old and tylosin (100 ppm in feed) from 8 1/2 weeks old until slaughter. During the partial depopulation, the dry sows received 1500 ppm chlortetracycline and 500 ppm tiamulin in-feed, whilst the nursing sows received 1875 ppm chlortetracycline and 625 ppm tiamulin from W30 to W36 (black coloured points).

Temporal fluctuations in AMR gene copy number and bacterial load (16S rRNA gene) can be observed in both the young pig (Fig. 2a) and dry sow (Fig. 2b) accommodation. Time point was a statistically significant explanatory variable for the 16S rRNA gene and all five AMR genes quantified in this study (Table 1). In the dry sow accommodation, high levels of AMR genes were detected in the absence of group antimicrobial administration.

**Table 1:** Summary of statistical model outputs (F-statistics and P-values) for assessing temporal shifts in gene abundances and microbiome diversity.

	Dry sow accommodation		Young pig accommodation	
	F-statistic	P-value	F-statistic	P-value
*tetB*	12.16	<0.001	2.05	0.030
*tetQ*	11.83	<0.001	5.60	<0.001
*ermA*	7.19	<0.001	2.01	0.034
*ermB*	30.02	<0.001	8.94	<0.001
*dfrA1*	20.69	<0.001	2.59	0.006
16S rRNA	13.30	<0.001	4.84	<0.001
ISI	5.63	<0.001	1.79	0.065
SI	5.79	<0.001	1.78	0.065

ISI = Inverse Simpson.

Index. SI = Shannon Index.

Prior to weaning and during co-housing (W1-W4), the levels of all five AMR genes were similar when comparing the nursing sow and young pig samples, in the presence of similar faecal bacterial load (Fig. 2a). During acidified water and zinc administration, an increase in *dfrA1* counts occurred in the young pig accommodation between W5 (mean 4.17 log_10_ copies/g DM, standard error of the mean (SEM) 0.80) and W7 (mean 5.58 log_10_ copies/g DM, SEM 0.65), and a decrease in *ermA* between W5 (mean 6.83 log_10_ copies/g DM, SEM 1.44) and W7 (2.70 log_10_ copies/g DM, SEM 1.17). There were no marked changes in the levels of *tetB, ermB* and *tetQ* during acidified water and zinc administration.

Chlortetracycline administration (W8-W10) was associated with a decrease in *tetB* gene copy number in the young pig accommodation between W9 (mean 6.95 log_10_ copies/g DM, SEM 0.79) and W10 (mean 3.29 log_10_ copies/g DM, SEM 0.79) with no significant changes in *tetQ, ermA, ermB or dfrA1* levels being observed during chlortetracycline administration. Tylosin administration (W11-W25) did not have an effect on AMR gene counts in the young pig accommodation following withdrawal from chlortetracycline. Increases in the majority of AMR genes being observed were seen from W21 to W24 in both the young pig and dry sow accommodation, which coincided with an increase in bacterial load, suggesting that this increase was independent of antimicrobial administration (see Supplementary Materials 3 for detailed statistical model outputs).

### AMR gene abundances and microbial diversity

**Figure 3:**
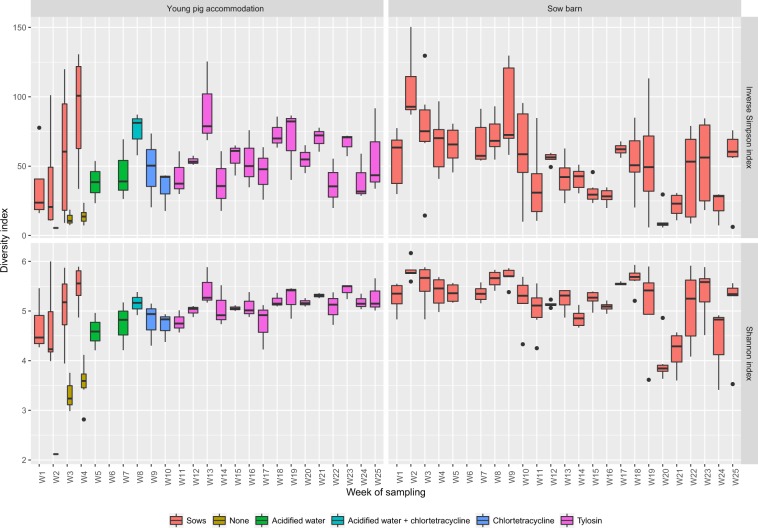
Alpha diversity indices of the faecal microbiota. The Inverse Simpson and Shannon indices of the faecal microbiomes obtained from the young pig accommodation and dry sow accommodation. On W1, faecal samples were obtained from pregnant sows only (n = 6) and on W2–4, faecal drops were obtained from both nursing sows (n = 6) and nursing pigs (n = 6) during co-housing.

16S rRNA gene metabarcoding was carried out to provide context on faecal microbiota development in the young pigs and whether changes in alpha diversity were linked to AMR gene abundances. Notable stepwise increases in measures of alpha diversity (both SIs and ISIs) occurred during the first 4 weeks of life (Fig. 3), highlighting an expected rapid increase in microbiota complexity. Even in faecal samples with low alpha diversity, comparable numbers of AMR genes were observed when comparing to nursing sow samples (Fig. 2a) and dry sow accommodation samples (Fig. 2b).

### AMR gene diversity over the production cycle

**Figure 4:**
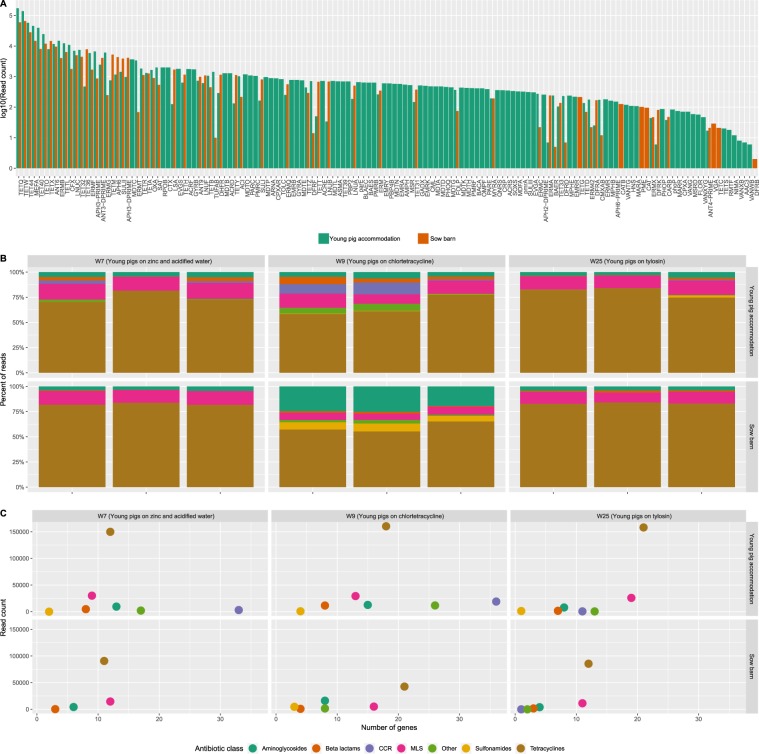
AMR gene abundance and diversity described by metagenomic data. (**A**) Log-transformed normalised gene abundance counts from both the dry sow and young pig accommodation (each bar is the sum of nine samples), (**B**) relative abundances of genes conferring resistance to particular antibiotic classes and (**C**) the sum of the absolute read counts from the three replicate samples conferring resistance to particular antibiotic classes.

A total of 144 AMR genes were detected on the farm (Fig. 4a), 21 of which were ubiquitous across sampling location and time point. The majority of the 60 genes that were present in both locations were more abundant (however, in the same order of magnitude) in the young pig accommodation. There was noticeably higher AMR gene richness within the young pig accommodation compared to the dry sow accommodation, with 77 AMR genes found solely in the former, compared to just 7 genes that were unique to the latter.

When examining the proportion of reads associated with each antibiotic class, AMR genes associated with tetracycline, macrolide (MLS) and aminoglycoside resistance predominated across all samples (Fig. 4b). With a few exceptions, these proportional results were consistent across the triplicate biological replicates. These proportional results were also broadly similar for the dry sows and young pigs on and between W7 and W25, despite the fact that the young pigs received tylosin from weeks 11 to 25.

The biggest change in the proportion of reads associated with each antimicrobial class occurred on W9 in both sampling locations. In the young pig accommodation, there was a proportional increase in reads associated with cross-class resistance (CCR). In the dry sow accommodation, the increase was seen in reads associated with aminoglycoside and sulphonamide resistance. These proportional changes were accompanied by an increase in AMR genes, from 94 genes in the young pig accommodation on W7 to 120 genes on W9 and 32 genes in the dry sow accommodation on W7 to 60 genes on W9. The proportion of reads associated with tetracycline resistance declined between W7 and W9, despite the young pigs starting chlortetracycline treatment during this time.

### AMR gene diversity and antimicrobial treatment

In the young pig accommodation, there were both high levels and numbers (i.e. >30) of genes conferring cross-class resistance (CCR) during zinc and acidified water (W7) and chlortetracycline administration (W9). This number dropped by the end of the production cycle, despite the young pigs having spent several months on in-feed antibiotics (Fig. 4c). Genes associated with CCR were not detected in the dry sow accommodation on W7 and W9, with only a single gene detected on W25. Nearly half i.e. 35 of the 77 genes found solely in the young pig accommodation were associated with CCR and appeared to be related to acidified water and zinc administration, rather than in-feed antibiotic (chlortetracycline and tylosin) administration.

### AMR gene quantification during and after a partial depopulation

To improve the health status of the farm, all the young pigs were removed (partial depopulation) and the sows treated with in-feed chlortetracycline and tiamulin. During this time period, all the pigs on the farm were treated with antibiotics simultaneously. During the last week of treatment (W36), and two (W45) and five (W57) months after cessation of treatment, bacterial load and AMR gene copy number in these samples did not change in response to the partial depopulation and antibiotic treatment (Fig. 2b).

## Discussion

When designing this study, our first hypothesis was that there would be a reservoir of AMR genes within the faecal microbiome, which would increase in both abundance and diversity in response to in-feed antimicrobial administration. Furthermore, we anticipated that AMR gene abundance would be inversely related to faecal microbiome diversity. Whilst large cross-sectional studies have been instrumental in furthering our understanding of AMR gene abundance and diversity across different livestock systems^12,21,35^, they have not provided the granularity of data and medicines usage history required to test these hypotheses. For this reason, we chose to undertake a longitudinal dissection of a single commercial pig unit, which included three group in-feed antibiotic treatment regimens and a partial depopulation event.

### Measured AMR genes consistently high across the unit

Although previous work has demonstrated high baseline levels of specific AMR genes^36^ and phenotypic resistance in *Escherichia coli* isolates^37,38^ in unmedicated pigs, our initial expectation was that AMR gene abundances would markedly increase in response to antimicrobial administration as a result of selective pressure being exerted on specific genes^39^. In agreement with previous work, we showed high levels of all studied AMR genes in the non-medicated dry sows, presumably as a consequence of the strong selection pressure exerted by this farm’s history of high antimicrobial usage. The levels of the five AMR genes studied in the young pigs reflected those of the sow population, whilst prolonged exposure to two different in-feed antibiotic regimens in the young pigs and a combined in-feed antibiotic regimen in the dry sows had no marked effect on AMR gene counts, potentially suggesting that they have reached saturation within the faecal bacterial populations^39^. Curiously, the antibiotics used (chlortetracycline and tylosin) were still clinically effective on this farm, suggesting that despite a high abundance of resistance genes within the faecal microbiome, that they were not present, or at least not active, within the organisms of clinical interest, i.e. *Mycoplasma hyopneumoniae, Mycoplasma hyorhinis, Actinobacillus pleuopneumoniae and Lawsonia intracellularis*.

Interestingly, some of the targeted AMR genes (i.e. *tetQ* and *ermB*) were present in higher copy numbers than the 16S rRNA gene at the majority of the studied time points. This is consistent with the work of other groups, which has demonstrated overall AMR gene abundances of 0.77–5.14^40^ and 0.54–3.1 gene copies^41^ per 16S rRNA gene in agricultural faecal and wastewater samples. It is worth noting that these AMR gene copy numbers are orders of magnitude higher than in samples obtained from other environments, such as sediments, soil and river water^41,42^. Collectively, these findings suggest that some bacteria are carrying multiple copies of particular AMR genes.

### Changes in AMR gene abundances were not associated with antimicrobial exposure

Week-to-week fluctuations in AMR gene abundance in both young pigs and dry sows were apparent, however the most marked changes were generally not associated with antimicrobial administration. In fact, the largest increases in AMR gene abundance were seen across all genes and in both classes of stock at the same time (W21-W24) which coincided with an increase in 16S rRNA gene copies, hence suggesting an as yet undefined environmental influence on bacterial load and consequently AMR gene abundance. A previous cross-sectional study highlighted that only 10–42% of the variation in AMR gene levels could be explained by factors included in statistical models (including lifetime antimicrobial exposure), suggesting that AMR gene levels are strongly influenced by a variety of other elements^43^. Whilst these could be related to feed changes, the fact that the dry sows and young pigs were housed differently and fed different diets^44^ would suggest that this effect is due to some other factors affecting the entire farm, such as housing and management^35,45,46^, environmental conditions^46^ or the introduction of an infectious agent.

### Microbiome alpha diversity was not affected by antimicrobial exposure

Similar to the studied AMR genes, fluctuations in microbiome alpha diversity were apparent in both dry sow and young pig accommodation samples. These changes were clearly more pronounced in the young pigs, as the alpha diversity of the faecal microbiome increased from nursing to finishing, which has been shown previously^47,48^. However, changes in the microbiome associated with antimicrobial treatment were not observed. Previous work has demonstrated that sub-therapeutic administration of chlortetracycline and tylosin had no impact on alpha diversity indices^47,49^. Whilst changes in relative abundances of specific taxa have been observed in response to tylosin administration, it was previously reported that these shifts were temporary, suggesting that the gut microbiota post-weaning seems to be resilient to perturbation by antimicrobial agents^47^. Despite using markedly higher levels of chlortetracycline and tylosin (300 ppm and 100 ppm, respectively), our results also showed that antimicrobial treatment did not impact on microbiome diversity in the young pigs.

### AMR gene abundances are high in nursing piglets with low microbiome diversity

With respect to our second hypothesis, even when the young pig faecal microbiome was at its least diverse during the suckling period, the AMR gene levels were comparable to that of the nursing sows. Although microbiome diversity increased dramatically in the piglets during the first few weeks of life, as reflected in previous studies^47,50–53^, this was not associated with changes in AMR gene prevalence. We expected, as others have proposed, that changes in the microbiota would influence AMR gene levels^20,44,54^, but in fact the high levels of studied AMR genes in the young pigs reflected that of the farm’s sow population. The most obvious explanation for this is a combination of vertical and horizontal transmission of bacteria at or shortly after birth^55,56^. The presence of comparable levels of AMR genes in both sows and piglets and the associated large differences in microbiome diversity suggest that the AMR genes studied appear to either be widespread across multiple taxa or highly concentrated within dominant taxa present throughout all stages of microbiota development.

### High AMR gene diversity and cross-class resistance genes in young pigs

Metagenomic sequencing revealed a diverse set of AMR genes in the presence and absence of antimicrobial treatment, which reflects findings in other recent work^19^. Specifically, genes associated with tetracycline and macrolide resistance predominated. This is not surprising given the history of high levels of tetracycline and macrolide use on this farm. Reassuringly, there was no evidence of co-selection between antimicrobial classes following chlortetracycline or tylosin administration. This is a significant finding and relevant to the principles of antimicrobial stewardship, where veterinary surgeons are actively discouraged from using fluoroquinolones and 3^rd^/4^th^ generation cephalosporins, so as to minimise the risk that resistance to these critically important antibiotics in human medicine is selected for in livestock.

What was evident, were the large numbers of genes associated with cross-class resistance (CCR) in the young pig accommodation samples taken on W7 and W9 and how these reduced by W25 and were almost absent from the unmedicated sows. The presence of these genes was not associated with the administration of chlortetracycline and tylosin in this study. Although the current study design does not allow us to disentangle temporal effects versus the effect of different treatments, it is interesting to note that the CCR genes were already highly abundant at W7, following a period of in-feed zinc administration and the use of acidified water. Copper and zinc salts are commonly administered in-feed at supranutritional levels due to their antimicrobial properties, with increasing doses of zinc oxide being previously shown to increase the abundance of both tetracycline and sulphonamide resistance genes in weaned pigs^57,58^.

The metagenomic sequencing results for the dry sow accommodation on W9 were unexpected. All three biological replicates demonstrated an increase in the proportion of reads associated with sulphonamide and aminoglycoside resistance. The richness of AMR genes also doubled at this time point compared to W7, despite no antimicrobial administration to this group of pigs. An increase in AMR gene diversity was also seen in the young pig accommodation at the same time point, however the increase was seen in beta-lactam resistance genes, CCR genes and genes associated with other antimicrobial classes. This is unlikely to be an artefact, given that this observation was seen across two different locations at the same time and five of the six biological replicates. This shift appears to be a consequence of an undefined environmental factor affecting the whole farm, where the two groups of pigs respond differently with regards to faecal AMR gene diversity.

### Persistently high abundances of AMR genes after partial depopulation

The partial depopulation at the end of the study involved considerable antibiotic use, with every sow on the farm receiving in-feed tiamulin and chlortetracycline. The treated sows were followed for five months after this treatment, during a period where antibiotic use on the farm declined nearly twenty-fold. Despite such dramatic changes in antibiotic usage, there were no marked changes in AMR gene abundances. This therefore begs the question as to how long (if at all) it would take to see reductions in AMR gene levels following the reduction or cessation of antimicrobial use on farms with a previous history of high-level use. We propose that the long-term use of these treatments has led to wide and stable dissemination of relevant resistance genes throughout the gastrointestinal microbial population of the pigs.

## Conclusion

AMR gene abundance and diversity on this unit were high in both dry sows and young pigs, likely as a consequence of historic antimicrobial usage. In this context, in-feed antibiotic administration (chlortetracycline and tylosin) did not affect faecal AMR gene abundance or diversity, suggesting that genes under positive selection in the presence of these antibiotics have already become stably integrated into the faecal microbiome. This would also potentially explain the failure of AMR gene levels to decline following dramatic reductions in antimicrobial use on the farm. Acidified water and zinc supplementation were linked to an increase in AMR gene diversity, which importantly included CCR genes, which decayed quickly after their withdrawal. This study did not identify which bacteria carried these AMR genes or indeed which genes were being expressed. Future work therefore needs to determine in what organisms these genes are not only present, but also active.

## Supplementary information

Additional methodology.

## Data Availability

The sequence data sets generated and analysed during the current study are publicly available on the NCBI Sequence Read Archive (16S rRNA gene metabarcoding data - BioProject accession number PRJNA557844) and the European Nucleotide Archive (shotgun metagenomic data - Study accession number PRJEB34736).
