# Supplementary material for: Resistance to change: AMR gene dynamics on a commercial pig farm with high antimicrobial usage
Source: Sci Rep. 2020 Feb 3;10:1708. doi: 10.1038/s41598-020-58659-3 (PMC6997390; doi:10.1038/s41598-020-58659-3)
Supplement: Supplementary file 1 — Additional methodology. [file 41598_2020_58659_MOESM1_ESM.pdf]

Supplementary Materials

Resistance to change: AMR gene dynamics on a commercial pig farm with high antimicrobial usage

Jolinda Pollock[1,2], Adrian Muwonge[2], Michael R. Hutchings[1], Geoffrey Mainda[2], Barend M. Bronsvoort[2], David L. Gally[2] and Alexander Corbishley[2]*

[1]Animal and Veterinary Sciences, Scotland's Rural College (SRUC), Edinburgh, United Kingdom

[2]The Roslin Institute and Royal (Dick) School of Veterinary Studies, University of Edinburgh, Edinburgh, United Kingdom

*Corresponding Author: Alexander Corbishley – alexander.corbishley@roslin.ed.ac.uk

Supplementary Materials 1 – Additional methodology

Herd health status and vaccinations

The herd was known to be positive for the following diseases: Porcine Reproductive and Respiratory Syndrome (PRRS), *Mycoplasma hyopneumoniae*, *Mycoplasma hyorhinis*, *Lawsonia intracellularis*, *Actinobacillus pleuopneumoniae*, *Streptococcus suis* and *Haemophilus parasuis*. Vaccines were used to control the following diseases: PRRS, erysipelas, parvovirus, clostridial disease, *Mycoplasma hyopneumoniae*, Porcine Circovirus Type-2 (PCV2) and enterotoxigenic *Escherichia coli*.

Production and housing information

During the study period, a mean of 12.9 piglets were born alive per sow, with mortality to weaning of 11.1%. Batch farrowing occurred every four weeks, with the study starting one week prior to the October 2016 batch farrowing. Nursing sows and piglets were housed on slats, whilst dry sows were housed in straw yards.

Historic antimicrobial administration

The following quantities of antimicrobials were used during the three months prior to the study period starting: 265.8 mg/PCU tylosin, 103.2 mg/PCU chlortetracycline, 9.0 mg/PCU dihydrostreptomycin, 5.6 mg/PCU benzylpenicillin, 3.4 mg/PCU amoxicillin, 1.3 mg/PCU lincomycin, 0.4 mg/PCU marbofloxacin, 0.3 mg/PCU oxytetracycline and 0.1 mg/PCU enrofloxacin. Previous batches of piglets prior to the study period received an additional in feed antimicrobial treatment of trimethoprim (100 ppm feed) and sulfadiazine (500 ppm feed) between 4 and 6 weeks old to control *Streptococcus suis* and *Haemophilus parasuis*.

Current antimicrobial administration

During the study period, the following routine group medication regimens were used: toltrazuril (30 mg/head oral) at 4 days old to control *Isospora suis*, zinc (2500 ppm in feed) between 4 and 6 weeks old to control post-weaning colibacillosis, acidified water (Baynes Evacide S 0.2%) to control post-weaning colibacillosis between 3 and 7 weeks old, chlortetracycline (300 ppm in feed) from 6 to 8 1/2 weeks old to control *Mycoplasma hyopneumoniae* and *Mycoplasma hyorhinis* and tylosin (100 ppm in feed) from 8 1/2 weeks old until slaughter to control *Mycoplasma hyopneumoniae*, *Mycoplasma hyorhinis*, *Actinobacillus pleuopneumoniae* and *Lawsonia intracellularis*.

During the partial depopulation, the dry sows received 1500 ppm chlortetracycline and 500 ppm tiamulin in-feed, whilst the nursing sows received 1875 ppm chlortetracycline and 625 ppm tiamulin. In the three months that included this partial depopulation, total antimicrobial use increased to 582.8 mg/PCU, which then declined to 32.3 mg/PCU in the three months after the partial depopulation.

Faecal sampling

On W1, faecal drop samples were collected from the floor of six farrowing crates containing pregnant sows (n = 6). Between W2 and W4, both sow and piglet faecal drop samples were obtained from the same farrowing crates as W1. On W5, all piglets were weaned and mixed into three groups prior to movement into the weaning house. From W5 to W13, pooled faecal drop samples were taken from each of these three pens to capture pen-level dynamics. On W14, these pigs were moved into the grower/finisher house and remained in the same pen formation as in the weaner house. Thereafter, from W14 to W25, pooled faecal drop samples were taken weekly from each of these pens until slaughter.

Quantitative PCR selection

The selection of AMR genes for targeted quantification (n = 5) was also based on the results of an initial end-point PCR screening of a sub-sample of DNA extracts obtained from the final sampling point (faecal samples = 6). A panel of 30 genes were selected, on the basis that these genes were of biological relevance to the historic use of antimicrobials on the pig unit and of importance to both veterinary and

human medicine (see **Supplementary Materials 2** for list of target genes and primer sequences). Genes which were amplified from more than 50% of the faecal samples were shortlisted for qPCR analysis.

Quantitative PCR methodology

qPCR mixtures were set up using Brilliant III Ultra-Fast qPCR Mastermix (Agilent Technologies, United States), reference dye (Agilent Technologies, United States) and the primers and probes listed in Supplementary Material 2. Each reaction was carried out in triplicate in a final volume of 20 µl, containing 1 µl of extracted DNA. Twenty-four samples were run per 96-well plate, which also included DNA standards (at concentrations ranging from 10^7 to 10^1 gene copies per µl) and a no-template control (nuclease-free water). Absolute quantification was carried out using a Stratagene MX3005P qPCR System (Agilent Technologies, UK) using the following fast, two-step cycling conditions: 95°C (5 minutes), followed by 40 cycles of amplification at 95°C (15 seconds) then 60°C (30 seconds).

Standard curves were constructed from the threshold cycle (C_T) values using the Stratagene MxPro Software (Agilent Technologies, UK) and within this software, the calculated gene copy number per µl for each of the samples was generated, treating each of the three replicates individually. Any samples which fell beneath the limit of detection (i.e. 10^1 copies per µl DNA, equivalent to 3.3 – 4.9 $\log_{10}$ copies/g DM) were re-run to confirm the findings. These values were then exported in Microsoft Excel spreadsheet format and the arithmetic means for the technical replicates calculated. These values were then converted into gene copy number per gram of dry matter (using the % DM values calculated) and $\log_{10}$-transformed for data visualisation and statistical analysis.

16S rRNA gene metabarcoding

Six library pools were compiled using equimolar concentrations of DNA from each sample. A mock bacterial community (20 Strain Even Mix Genomic Material ATCC®MSA-1002, ATCC, United States) and a reagent-only control (generated by passage of nuclease-free water throughout the library preparation process) were included in each pool to assess background contamination and sequencing

error rate. Using the mock bacterial community sequences, the mean sequencing error rate was calculated as 0.01%.

The pools were submitted to the sequencing centre (Edinburgh Genomics, United Kingdom) where the pools were quantified using the Quant-iT™ PicoGreen® double-stranded DNA Assay Kit (Thermo Fisher Scientific, UK) to ensure sufficient yield for sequencing. Sequencing was carried out using the Illumina MiSeq platform (Illumina, United States), using V2 chemistry and producing 250 bp paired-end reads.

Metagenomic sequencing

Illumina TruSeq DNA Nano libraries were prepared using the submitted faecal DNA extracts and sequencing was carried out using the HiSeq 4000 platform generating 150 bp paired-end reads (Illumina, United States).

Host DNA was removed from the raw reads by mapping to the *Sus Scrofa* reference genome GCA_000003025 version 11.1 and Phix DNA (PhiX 174) was removed using the run_contaminant_filter.pl script which is part of the Microbiome Helper suite. Read quality control was carried out using trimmomatic. These paired end reads were then mapped to MEGARes, a hand-curated AMR gene database, using the paired-end option of BWA. The resistome profiling was carried out using the ResistomeAnalyzer function in MEGARes with the gene fraction threshold set at 90. In order to obtain a read per kilobase (RPK) normalised abundance count, the Humann2 script which is part of the Humann2 pipeline was used to analyse the SAM file produced from the mapping step above. The resultant gene family output was then normalised using the humann2_renom_table script. The generated tsv and csv files were then used to analyse AMR gene abundances in R version 3.5. The downstream analysis of AMR gene diversity and data visualisation were carried out using the ggplot2 package in R.

Supplementary Materials 2 – Primer and Probe Sequences

Target	Forward Primer	Reverse Primer	Probe	Size (bp)	Annealing temp used °C)	Reference
tetA	GCTACATCCTGCTTGCCTTC	CATAGATCGCCGTGAAGAGG		210	55	Ng et al (2001)
tetB	TTGGTTAGGGGCAAGTTTTG	GTAATGGGCCAATAACACCG		659	55	Ng et al (2001)
tetC	CTTGAGAGCCTTCAACCCAG	ATGGTCGTCATCTACCTGCC		418	55	Ng et al (2001)
tetM	ACAGAAAGCTTATTATATAAC	TGGCGTGTCTATGATGTTCAC		171	55	Aminov et al (2001)
tetO	ACGGARAGTTTATTGTATACC	TGGCGTATCTATAATGTTGAC		171	60	Aminov et al (2001)
tetQ	AGAATCTGCTGTTTGCCAGTG	CGGAGTGTCAATGATATTGCA		169	63	Aminov et al (2001)
tetS	GAAAGCTTACTATACAGTAGC	AGGAGTATCTACAATATTTAC		169	50	Aminov et al (2001)
tetT	AAGGTTTATTATATAAAAGTG	AGGTGTATCTATGATATTTAC		169	50	Aminov et al (2001)
tetW	GAGAGCCTGCTATATGCCAGC	GGGCGTATCCACAATGTTAAC		168	64	Aminov et al (2001)
otrA	GGCATYCTGGCCCACGT	CCCGGGGTGTCGTASAGG		212	66	Aminov et al (2001)
tetX	CAATAATTGGTGGTGGACCC	TTCTTACCTTGGACATCCCG		468	55	Ng et al (2001)
dfrA1	GTGAAACTATCACTAATGG	ACCCTTTTGCCAGATTTG		471	50	Seputiene et al (2010)
dfrA8	TTGGGAAGGACAACGCACTT	ACCATTTCGGCCAGATCAAC		382	50	Chen et al (2004)
dfrA12	GGTGAGCARAAGATYTTTCGC	TGGGAAGAAGGCGTCACCCTC		309	50	Seputiene et al (2010)
dfrA22	GCBAAAGGDGARCAGCT	TTTMCCAYATTTGATAGC		394	50	Seputiene et al (2010)
dfrB	GATCACGTRCGCAAGAARTC	GACTCGACVGCRTASCCTTC		95	56	Seputiene et al (2010)
sul1	CGGCGTGGGCTACCTGAACG	GCCGATCGCGTGAAGTTCCG		433	69	Kerrn et al (2002)
sul2	GCGCTCAAGGCAGATGGCATT	GCGTTTGATACCGGCACCCGT		293	69	Kerrn et al (2002)
sul3	GAGCAAGATTTTTGGAATCG	CATCTGCAGCTAACCTAGGGCTTTGGA		750	51	Perreten et al (2003)
ermA	TCTAAAAAGCATGTAAAAGAA	CTTCGATAGTTTATTAATATTAGT		645	52	Sutcliffe et al (1996)
ermB	GAAAAGGTACTCAACCAAATA	AGTAACGGTACTTAAATTGTTTAC		639	52	Sutcliffe et al (1996)
ermC	TCAAAACATAATATAGATAAA	GCTAATATTGTTTAAATCGTCAAT		642	51	Sutcliffe et al (1996)
ermF	CGACACAGCTTTGGTTGAAC	GGACCTACCTCATAGACAAG		309	50	Chen et al (2007)
ermG	ACATTTCCTAGCCACAATC	CGCTATGTTTAACAAGC		442	50	Shoemaker et al (2001)
blaCTX-M	CGATGTGCAGTACCAGTAA	TTAGTGACCAGAATCAGCGG		585	60	Castillo et al (2013)
blaCTXM-1	ATGGTTAAAAAATCACTGCG	TTACAAACCGTCGGTGAC		876	60	Castillo et al (2013)
blaCTXM-9	ATGGTGACAAAGAGAGTGCAAC	TTACAGCCCTTCGGCGATG		876	60	Castillo et al (2013)
blaCTXM-2	TCAAGAAGAGCGACCTGGTT	GATACCTCGCTCCATTTATTG		601	60	Castillo et al (2013)
qnrA	CAGCAAGAGGATTTCTCACG	AATCCGGCAGCACTATTACTC		630	63	Ciesielczuk et al (2013)
qnrB	GGCTGTCAGTTCTATGATCG	SAKCAACGATGCCTGGTAG		488	63	Ciesielczuk et al (2013)
qnrS	GCAAGTTCATTGAACAGGGT	TCTAAACCGTCGAGTTCGGCG		428	60	Cattoir et al (2007)

tetB	**TTACGTGAATTTATTGCTTCGG**	**ATACAGCATCCAAAGCGCAC**	**CGCCGACCAAATCGGTCAGA**	**206**	**60**	**Schmidt et al (2015)**
tetQ	**AATTACTGTTCGGGCTTCTA**	**GCTTGTATGCCTTCCTTTGC**	**ATCTATTATCTGGAATGGAGTGAAATGCAA**	**162**	**60**	**Maeda et al (2003)**
ermA	**AGTTCATTATAACCAGTAAGGAGAAGG***	**CGACTCATTTTGACTAGCTCTTTG***	**TGAACCAGAAAAACCCTAAAGACACGCA***	**190**	**60**	**This study**
ermB	**GGATTCTACAAGCGTACCTTGGA**	**TGGCAGCTTAAGCAATTGCT**	**CACTAGGGTTGCTCTTGCACACTCAAGTC**	**90**	**60**	**Böckelmann et al (2009)**
dfrA1	**ATGGAGTTATCGGGAATGGC***	**ACTTTCGGTTGGGTAATGCTC***	**AGGAGCTGTTCACCTTTGGCACT***	**141**	**60**	**This study**
16S rRNA	**ACTCCTACGGGAGGCAGCAGT**	**TATTACCGCGGCTGCTGGC**	**CGCGTGACCCTTATTGCTCCACA***	**194**	**60**	**Clifford et al (2012)**

* Designed as part of this study. Primers/probes were designed using Primer BLAST (NCBI), with the latter being checked for specificity against the GenBank nr database.

Bold type indicates primers and probes used for qPCR

Supplementary Materials 3 – Statistical outputs

Young pig accommodation: Assessment of temporal variation of mean gene copy number and mean diversity indices by analysis of variance, presented with the mean standard error of difference (SED) for each model. The models excluded samples from W1 (pens only contained pregnant sows at this time point) and samples from W2-W4 when the young pigs were still grouped by litter and were not yet assigned to the rearing pens.

Week	16S rRNA	*dfrA1*	*ermA*	*ermB*	*tetB*	*tetQ*	ISI	SI
5	9.736	4.168	6.827	9.021	6.577	9.735	39.16	4.579
7	8.591	5.577	2.702	8.598	6.240	9.068	44.94	4.736
8	8.971	6.244	4.968	8.606	6.433	9.386	75.45	5.156
9	8.678	5.535	4.561	8.434	6.952	9.242	48.18	4.797
10	9.546	5.561	3.597	9.162	3.293	9.578	34.46	4.716
11	9.212	5.660	5.266	9.173	3.782	9.519	42.75	4.777
12	9.040	5.211	5.063	8.746	5.891	9.420	53.82	5.008
13	8.295	3.371	3.032	8.106	3.838	8.465	91.04	5.442
14	8.829	6.992	6.694	8.235	6.497	8.461	38.11	5.057
15	8.431	5.947	3.238	8.224	5.525	8.729	56.37	5.056
16	8.667	5.746	3.180	8.491	5.359	8.890	53.61	5.092
17	8.494	5.759	1.798	8.282	5.944	8.873	45.84	4.754
18	9.789	5.473	4.464	8.665	5.092	9.045	72.84	5.204
19	8.989	5.879	3.545	8.693	5.524	9.134	69.58	5.240
20	8.987	6.190	5.077	8.587	6.190	9.088	55.04	5.170
21	9.047	5.484	0.006	8.723	5.680	9.198	70.13	5.311
22	10.344	7.384	6.167	9.794	6.930	10.290	36.93	5.076
23	10.979	7.663	4.187	11.373	7.517	10.659	66.67	5.419
24	10.777	7.707	5.798	10.545	7.310	10.335	39.79	5.178
25	8.523	6.974	5.531	10.917	5.787	10.272	56.26	5.274
Mean SED	0.492	0.928	1.670	0.443	1.136	0.376	16.32	0.757

Sow barn: Assessment of temporal variation of mean gene copy number and mean diversity indices by analysis of variance, presented with the mean standard error of difference (SED) for each model.

Week	16S rRNA	*dfrA1*	*ermA*	*ermB*	*tetB*	*tetQ*	ISI	SI
1	10.090	5.558	7.190	8.618	5.329	9.574	55.4	5.278
2	9.102	6.986	6.646	8.020	6.984	8.987	106.2	5.851
3	10.000	7.088	7.455	8.860	6.899	9.691	75.9	5.541
4	9.723	4.868	5.929	7.635	5.515	9.459	66.5	5.387
5	9.552	5.546	5.819	8.240	5.855	9.345	64.9	5.362
7	9.282	5.359	6.682	8.363	5.598	9.191	67.1	5.352
8	9.361	7.760	6.985	8.365	7.506	8.323	72.0	5.644
9	9.489	7.736	6.827	8.273	7.112	8.151	90.2	5.692
10	9.219	6.522	7.143	8.884	5.523	9.267	60.2	5.221
11	10.123	7.634	7.907	8.849	7.331	9.436	36.5	5.022
12	8.657	5.368	6.952	8.477	4.624	8.870	55.8	5.134
13	9.065	6.562	7.057	8.009	6.784	8.433	41.9	5.236
14	9.083	6.084	6.712	8.435	6.008	9.134	41.0	4.857
15	8.910	7.520	6.645	7.632	6.794	7.925	31.4	5.248
16	8.845	5.811	6.615	8.487	5.603	8.640	28.3	5.084
17	8.760	7.771	6.606	8.067	7.025	8.209	61.6	5.550
18	10.109	8.570	7.152	8.327	7.593	9.085	54.2	5.650
19	9.213	7.616	7.086	8.007	6.756	8.634	54.5	5.085
20	7.987	7.506	6.352	7.728	5.248	7.273	11.4	3.980
21	8.468	5.072	6.155	8.314	2.783	8.340	21.9	4.187
22	10.704	8.960	7.572	9.757	8.211	9.278	44.7	5.073
23	10.791	8.420	8.044	11.611	7.607	10.263	52.9	5.382
24	10.572	9.741	7.779	10.176	8.663	8.600	21.8	4.384
25	8.736	7.404	7.039	9.725	6.007	9.708	55.2	5.094
Mean SED	0.283	0.408	0.301	0.234	0.517	0.279	13.10	0.304
